# A rare cause of small bowel perforation: A metastatic lesion from squamous cell carcinoma of the tongue

**DOI:** 10.1016/j.ijscr.2020.02.011

**Published:** 2020-02-07

**Authors:** Hadeel AlOmran, Layla AlBayyat, Hisham AlMiman, Deena Boqari, Mohammed AlDuhileb, Tariq Madkhali

**Affiliations:** a Department of General Surgery, King Fahad Specialist Hospital, Dammam, Saudi Arabia; b Department of Pathology, King Fahad Specialist Hospital, Dammam, Saudi Arabia

**Keywords:** Tongue, Sequamous, Carcinoma, Metastasis, Gastrointestinal, Perforation

## Abstract

**Background:**

Metastatic lesions from head and neck tumours to the small bowel are extremely rare, and metastasis from tongue squamous cell carcinoma in particular has been reported only twice in the English literature.

**Case presentation:**

A 76 year-old lady diagnosed with a loco-regionally advanced tongue squamous cell carcinoma but deferred surgical resection. Seven months later after her diagnosis, she presented with generalized abdominal pain, due to image-proven viscous perforation, thus taken for emergent exploratory laparotomy, where the perforation was found at the distal ileum, which was resected, and a primary side to side anastomosis was done. The histopathological examination of the resected segment revealed squamous cell carcinoma originating from the tongue. The patient was deteriorating clinically and vitally during her stay, and passed away on day 16 post operation due to multi-organ dysfunction syndrome.

**Conclusion:**

Metastatic lesions from tongue carcinoma found in the vicinity of the small bowel represent a terminal stage with a dismal prognosis, and when these lesions present clinically with a complicated course, the survival rate is decreased significantly.

## Highlights

• Metastasis from head and neck tumors to the gastrointestinal tract is rare.
• The mechanism of metastasis in those cases is unclear due to the rarity.
• Based on the existing data, prognosis is dismal in such cases.
• Perforation of the bowel can happen either spontaneously due to tumor erosion of the wall, or it can be induced by chemotherapy.

## 1 Introduction

Metastatic lesions from head and neck tumours to the small bowel are extremely rare [1,2]. Metastatic lesions found in the vicinity of the small bowel represent a terminal stage with a dismal prognosis, and when these lesions present clinically with a complicated course, i.e. perforation, the survival rate is decreased significantly [2]. In this paper, we present a case of a 76 year-old lady who was diagnosed with a loco-regionally advanced tongue squamous cell carcinoma but deferred surgical resection. Seven months later after her diagnosis, she presented with a perforated small bowel. The histopathological examination of the resected segment revealed squamous cell carcinoma originating from the tongue. This case was reported in line with SCARE criteria [3].

## 2 Case presentation

Our patient is a 76 year-old lady, who has been diagnosed with a loco-regionally advanced, stage IVb (T3N2cM0) squamous cell carcinoma of the tongue in September 2018. Surgical resection was offered to her early in the course of her disease, but she refused it. She was on a regular follow up with our medical and radiation oncology facilities, and since she was deemed unfit for systemic therapy, she was managed with radical loco-regional radiation therapy, 70 Gy/35 fractions, and she finished her course in December 2018, with a partial response, with an eventual development of pulmonary metastasis that was detected on follow up imaging in January 2019.

**Fig. 1:**
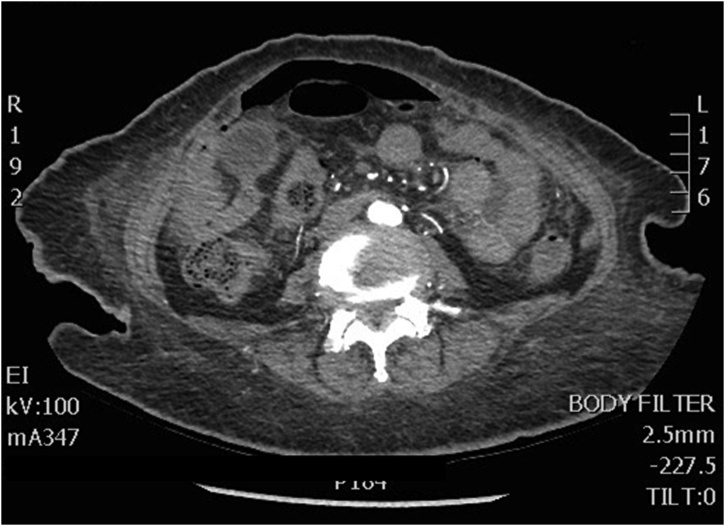
Plain Computed Tomography scan of the abdomen and pelvis showing moderate amount pneumoperitoneumwith thickening of the peritoneal reflections; consistent with viscous perforation.

**Fig. 2:**
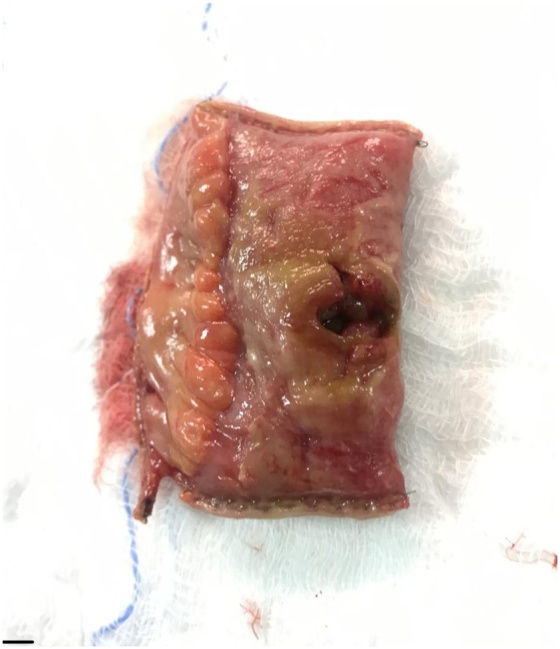
A gross picture of the resected specimen showing a perforated area at the anti-mesenteric wall of an ileal segment with necrotic appearance.

**Fig. 3:**
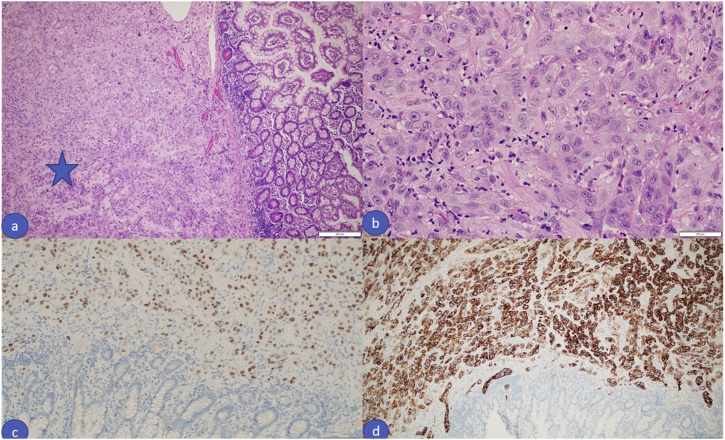
Histopathology examination of the resected segment (a) Microscopic examination revealed infiltration of the small bowel muscular wall by diffuse sheets of neoplastic cells (Star mark), H&E, 4x, (b) Higher magnification showing poorly differentiated neoplastic cells with some intra-tumor neutrophilic infiltrate, H&E, 40x, (c) Immunohistochemical staining showing the neoplastic cells staining positive for P63 and (d) CK 5/6.

On the 22nd of March 2019, the patient presented to the emergency department with altered mental status, progressively increasing generalized abdominal pain over a period of 3 days associated with subjective fever. She also had worsening of her chronic constipation, however, this wasn’t associated with nausea, vomiting, or abdominal distention. On examination, she looked dehydrated, confused and in severe pain. Her initial vital signs were: blood pressure 109/56 mmHg, heart rate 110 beats per minute, oxygen saturation of 99% on 4 L nasal cannula, and she was afebrile at that time. Her abdomen looked slightly distended, with guarding and tenderness on palpation. Her initial laboratory findings were: white cell count 12 × 10^12^/L, haemoglobin 12 g/dl, lactate 3 mmol/L, PH 7.33, pCO2 43 mmHg, and bicarbonate 21.1 mmol/L. Her ECG showed sinus tachycardia, and acute abdominal series revealed the presence of free air under the diaphragm. Computed Tomography scan was obtained, and revealed moderate pneumo-peritoneum, that appeared to be likely from a perforated proximal transverse colon, with a moderate amount of peri-hepatic and pelvic free fluid (Fig. 1). Computed Tomography images of the brain were unremarkable. On the premise of the diagnosis with a perforated viscus, the family was consented for exploratory laparotomy. Intra-operatively, a perforation at the distal ileum was found 30 cm away from the ileocecal valve, with no palpable masses in or near to the perforation site. The affected area was resected (Fig. 2), anda primary side-to-side anastomosis was done. The rest of the bowel was not affected. The patient was shifted to the intensive care unit being intubated. Histopathological examination was consistent with poorly differentiated squamous cell carcinoma that stained positive for CK7, CK5/6, and P63, and negative for CK20, and CDX2 (Fig. 3A–D). The surgical margins were also positive for the disease.

During her stay, she was steeply deteriorating, with increasing doses of inotropic medications. On day 16 post operatively, she developed bilateral pulmonary infiltrates with thick tracheal secretions, and need of higher ventilator settings, and her drain output was increasing; picture that was worrisome of ventilator associated pneumonia and anastomotic leak. On day 16 post operatively, she arrested, the cause of death was attributed to multi-organ failure disorder.

## 3 Discussion

Small bowel tumours are among the rare gastrointestinal tract malignancies with an incidence of 1 per 100,000 populations [1]. About 10% of those tumours are metastatic lesions arising from intra-abdominal malignancies, most notably the uterus, the cervix and the colon [2]. Metastasis from extra-abdominal tumours, including the breast, oesophagus, lung, and melanoma, are far less common, however, a good number of cases have been reported in the literature [2,4].

Incidence of distant metastasis from head and neck tumours ranges from 7 to 23%, most commonly to the lung, liver and bones, and less frequently to the skin and brain [2,6,7]. A very few cases have been reported where the primary lesion metastasized to the small bowel, which often is found to be laryngeal squamous cell carcinoma [2]. Moreover, metastasis to the small bowel from tongue carcinoma in particular has only been reported only twice so far (Table 1) [5,8]. Given their extreme rarity, metastasizing head and neck tumours to the small bowel are poorly understood.

**Table 1:** The clinical features of the published cases in the literature.

Author (year)	Age	Tumor stage	Loco-regional management	Metastasis regions	Perforated segment	Reason for perforation	Mortality
Aoyagi et al. (2011) [5]	40 year old	Stage IV	Neo-adjuvant chemotherapy, then subtotal laryngectomy, followed by radiotherapy	Recurrence as mediastina and abdominal wall lesions	Ileum	Chemotherapy-induced tumor necrosis	Not mentioned
Taneja et al. (2018) [8]	60 year-old	Stage IV	Palliative chemotherapy	Lungs	Ileum	Chemotherapy-induced necrosis	On day 10 post operation
Present case (2019)	76 year old	Stage IV	Radiation therapy	Lungs	Ileum	Spontaneous tumor necrosis	On day16 post operation

The mechanism of metastasis to the small bowel is due to spread of micro-metastatic lesions through the blood-stream, however, spread through the lymphatic system has not been proven [2,5]. Based on the analysis done by Diwividei et al. on 12 published cases of secondary small bowel tumors from the head and neck origin, ileum was found to be the most common site for metastatic lesions involvement, followed by the jejunum and in only one case the duodenum was involved [2]. In general, the predictors of metastasis from head and neck tumours include: the location of the primary tumour, tumour stage, lymph node involvement, and failure of control over the loco regional disease [6,7]. In a contemporary analysis of 141 patients with metastasizing head and neck tumours done by Duprez et al, hypo-pharyngeal tumours were the most common metastasizing lesions, which was in keep with the previous studies, whereas oral tumours, collectively, ranked third [7] though a controversy exists as some of the old studies found that oral tumours have less chance of metastasis compared to the other sites [9]. Looking on the other way around, Diwividei et al. found that secondary small bowel tumours from head and neck were most commonly laryngeal in origin, with predominance of supraglottic carcinomas [2]. Moreover, the likelihood of metastasis is higher with the higher T class; lymph node involvement is an important predictor of distant metastasis of head and neck tumours, as it’s rare for a metastatic lesion to be found in the absence of a regional lymph node involvement, and the rate increases significantly with N3 disease, 16.7% vs. 9.2% in N2 disease [2,6]. The risk of distant metastasis in stage I, II, III and IV head and neck tumours are 1%, 14%, 15% and 20%, respectively [6]. Our patient had multiple risk factors, namely advanced stage at diagnosis and failure of loco-regional control of the disease, and the subsequent pulmonary metastasis, all of which probably put her on the unique risk of developing small bowel metastasis, which represents a terminal stage of her illness.

Once distant metastasis is diagnosed with head and neck tumour, the prognosis is dismal, and the survival is less than 2 years in >90% of the patients and the median time from diagnosis till demise is +/− 4 months [6,7]. And in the case of our patient, death is inevitable since she didn’t only present with metastasis, but the bowel was also perforated, thus she succumbed to her disease within less than a month.

Secondary malignancies of the small bowel are rarely symptomatic, but when they are, the symptoms are dependent on the site involved. Those symptoms can either be vague and non-specific, which might not raise the suspicion of metastasis since it’s extremely rare, or they could be complicated. Complicated ileal lesions are often obstructive, whereas, perforation is mostly noted in the jejunum [2]. Perforation in secondary small bowel tumours has been attributed to a number of possibilities, including: necrosis of the mass into the bowel wall due to chemotherapy, ischemic changes caused by embolization of the metastatic lesions, and bowel wall rupture due to increased intra-luminal pressure [5]. The predictors of this complication are probably related to the histopathological features of the tumour including its grade, as well as the use of immune-suppressants, chemotherapy, and radiotherapy [2]. The cause of perforation in the two previously reported cases is chemotherapy-induced tumour necrosis; our patient didn’t receive any chemotherapeutic agent, thus, we rather believe that the tumor cell deposit caused spontaneous necrosis into the bowel wall.

There are no consensuses on the appropriate management of patients with small bowel metastasis from head and neck tumours, but in general the intents of any treatment modality is palliative [2]. Major surgical procedures are only warranted in emergency situations as in the cases of bowel obstruction and perforation; diverting stoma alone are better offered for the former, and partial resection with end stoma creation in the perforated cases provide a survival for a few weeks [2,5].

## 4 Conclusion

In conclusion, secondary small bowel malignancies from the head and neck tumours are extremely rare, especially those arising from tongue cancer. Due to their rarity, vague abdominal complaints are often overlooked until the disease takes a more complicated course such as obstruction or perforation. The presence of those metastatic lesions carries a very poor prognosis, and being coupled with bowel perforation, survival can be limited to only a few weeks.

## Sources of funding

No funding was required.

## Ethical approval

Case reports do not require ethical approval as per King Fahad specialist hospital protocol.

## Consent

A written, informed consent was obtained from the patient’s next of kin for publication of this case and the accompanying images as per KFSHD and IJSCR guidelines. A copy of the written consent is available for review by the Editor-in-Chief of this journal on request".

## Author contribution

**Concept built**: Mohammed AlDuhlieb and Hisham AlMaiman.

**Data collection**: Hadeel AlOmran and Mohammed AlDuhlieb.

**Manuscript writing**: Hadeel AlOmran.

**Manuscript review**: Mohammed AlDuhlieb.

**Pathology diagnosis**: Deena Boqari.

**Pathology picture preparation and description writing**: Layla AlBayyat.

**Surgeon and main responsible physician**: Tariq Madkhali.

## Registration of research studies

Not applicable.

## Guarantor

Hadeel AlOmran.

## Provenance and peer review

Not commissioned, externally peer-reviewed.

## Declaration of Competing Interest

We declare no conflict of interest.
